# The Role of Health Information in Consumers’ Willingness to Pay for Canned Crushed Tomatoes Enriched with Lycopene

**DOI:** 10.3390/nu11092173

**Published:** 2019-09-10

**Authors:** Fabio Verneau, Francesco La Barbera, Marilena Furno

**Affiliations:** 1 Department of Political Science, University of Naples Federico II, 80138 Naples, Italy; verneau@unina.it (F.V.); francesco.labarbera@unina.it (F.L.B.); 2 Department of Agricultural Sciences, University of Naples Federico II, 80055 Naples, Italy

**Keywords:** quantile regression, experimental auction, lycopene, information

## Abstract

The paper investigated whether information about the health benefit produced by lycopene could influence consumers’ willingness to pay (WTP) for canned crushed tomatoes enriched with lycopene. An additional aim was to determine whether the main socio-demographic variables, such as sex, age, income and selected attitudinal factors, affect WTP. To this end, a non-hypothetical experimental auction was carried on with five repeated rounds. Results show a relevant impact of information on WTP in the case of lycopene-enriched products, whereas no difference in bids emerges for the conventional product, either on average or at the quantiles. Previous knowledge seems to have a modest influence upon WTP, but it shows a significant interaction with the information shock provided during the experiment, so that the effect of the latter is more pronounced when previous knowledge is low. In addition, age, sex, food technology neophobia, trust in science and implicit attitudes towards food technology significantly affect participants’ WTP.

## 1. Introduction

Functional products are among the most interesting food categories in terms of market opportunities, and one of the faster growing food sectors. This global growth is strongly related to the increased consumers’ awareness about the role of food in wellbeing, either to help prevent chronic diseases, or to optimize health [1,2,3]. Moreover, due to the economic costs of the medical care of chronic diseases, authorities have developed actions and policies to promote the consumption of functional food [4]. Despite the considerable success and interest, there is not yet a universally accepted definition for this group of food [5]. On a very general level, they could be defined as food products providing an added health benefit over and above the traditional food nutritional value [6,7].

From the production point of view, functional food is an opportunity to face the increasing global competition and to boost a progressively more saturated food demand [8]. However, even if functional food could be a good firm strategy by creating differentiated, value-added products, many of those innovative products have failed, probably due to a lack of cooperation between the disciplines involved in the new product development process, especially those focusing on consumers’ attitudes and knowledge [9]. As a matter of fact, at least in Europe, the performance of new functional food launched on the market has not always been in line with the optimistic expectations of the producers, and the results in terms of profitability have been sometime disappointing. A controversial attitude towards functional products has been observed in Europe, where people are more reluctant in adopting functional food than in Asia or North America [8,10].

The last survey of the Spanish Government revealed that consumers attach more importance to the origin of the product (i.e., those produced in their own region, or with a Protected Denomination of Origin), than to functionality [11]. Moreover, Backstrom and colleagues [12], analyzing focus group discussions, report that functional food is associated with a medicine-like representation, evoking quite negative impressions among participants. In a similar fashion, Frewer, Scholderer, and Lambert [6] emphasize that consumers’ risk perceptions may have an important role in reducing any willingness to accept functional food. Finally, Furno et al. [13] performed experimental auctions on between samples in order to study hypothetical bias., they used as a product canned tomatoes enriched in lycopene, and highlighted ing the role of explicit and implicit attitudes towards food technology.

Due to the controversial attitudes of European consumers, the development of consumer-oriented new products becomes a crucial element to recover the required up-front investments, and more generally to generate adequate levels of profitability and the growth rate of companies. Indeed, the development of new functional components and their relative technological solutions can be very challenging and expensive. To avoid major failures in investments, it is therefore important to monitor consumers’ attitudes to ensure that new functional products fulfil consumer expectations, and that any health-related information is honestly and attractively communicated. In fact, knowledge and information have been demonstrated to exert a strong effect on consumers’ decision-making towards functional food. In their 1999 report of a qualitative study, the International Food Information Council (IFIC) [14] indicated that lack of knowledge is the major reason for not consuming functional food. Also, de-Magistris and colleagues [15] highlighted the crucial role of knowledge about nutrition in their study on the influence of body image on consumers’ willingness to pay (WTP) for potato chips. Several other studies indicated information and knowledge, both subjective and objective, as relevant antecedents of functional products’ consumption [16,17,18]. In addition to the intervening factors already emerged in previous studies, Temesi et al. [19] have found a new factor called the Perceived Correspondence of Health that plays an important role in consumers’ perception of functional ingredients.

However, most of the research on consumers’ acceptance of functional food, dealing with information and its effects on the willingness to pay (WTP) or accept, is limited to investigating whether information and/or different types of information may alter this WTP [20,21,22,23]. To the best of our knowledge, the study of the main factors influencing the change in the willingness to pay due to the administration of information is a topic still understudied.

The products chosen for the present study are canned tomatoes, one of the most important products of the food industry in southern Italy [24]. Tomatoes naturally contain lycopene, a carotenoid responsible for the red color which has received considerable attention due to its various biological activities [25]. As a matter of fact, several studies indicate lycopene among the main natural components capable of inhibiting the proliferation of cancer cells in some of the most common types of cancer [26,27]. Yet, it should also be noted that the European Food Safety Authority (EFSA) has not yet approved any health claims related to lycopene considering the scientific evidence still insufficient [28]. Given this not yet well-defined scenario on health claims related to lycopene, the focus of the present study is to investigate whether information about the possible health benefits produced by lycopene could influence consumers’ WTP for canned crushed tomatoes enriched with lycopene.

Our specific objectives are:

1. To determine whether information on the benefit of lycopene affects the consumers’ WTP both on canned tomatoes enriched with lycopene and conventional canned tomatoes (not enriched).
2. To analyze whether and how several selected variables, namely socio-demographics, attitudinal constructs and control variables, affect WTP. More in detail, the impact of these variables on WTP will be investigated also in the tails of the bid distribution;
3. To explore whether the selected variables affect the possible change in prices, after the informative shock; that is, assessing the moderator role of the variables on the WTP.

We undertook this study using data from an experimental laboratory auction conducted in Italy. Two treatments differing in the information provided to the participants were taken into consideration. This study expands the literature on consumers’ preferences for functional food products using the experimental auction method. Currently, the use of a non-hypothetical auction method has gained popularity in estimating the WTP for product attributes or new products [29], alongside a real Choice Experiment [15,30]. A major reason for the increasing popularity of experimental auctions is their incentive compatibility property, that is, subjects have the dominant strategy to submit bids equal to the true value of the goods. The experimental auction would then be demand-revealing, and hence the participants would provide truthful bids [31]. Indeed, in accordance with Chang, Lusk and Norwood [32], the WTP values from experimental auctions can be assumed to be the true values corresponding to the actual payments in the marketplace (i.e., scanner data from shopping); thus, they are a better approximation of the true preferences.

## 2. Materials and Methods 

### 2.1. Participants

A total of 100 participants were recruited for the auctions, selected among the college students from several departments of the University of Naples. All of the various steps of the experiment were computer administered; moreover some phases of the experiment, such as the Single Category Implicit Association Test (SC-IAT) text, require promptness and great attention. It is very likely that students have a greater ability to perform such tasks. In addition, Depositario et al. (2009) pointed out that prices and opinions on products issued by students are in line with those declared by responsible purchasers. [33] Therefore, the students’ answers in this experiment should not generate significant distortions. Main statistics on socio-demographic characteristics are presented in Table 1. Age is a numerical variable expressing the number of years of the participant’s life. Gender is a binary variable which assumes value 1 in the case of the female gender, and 0 in the case of the male gender. Income is a categorical variable which measures net monthly family income and assumes value 1 for incomes up to 1000 Euros; 2 for incomes between 1000 and 2000 Euros, 3 for incomes between 2000 and 3000 Euros, 4 for incomes over 3000 Euros.

### 2.2. Measures

In the late 1980s and early 1990s, as a response to widespread criticism against self-report measures, new instruments began to appear in social psychology for measuring implicit attitudes, that is, attitudes that are not completely conscious, and/or that are automatically activated. Research showed the relevant predictive validity of these new measures [34,35], also in the food domain [36,37,38]. Among the most widely used instruments for measuring implicit attitudes are the Implicit Association Test (IAT) [39], and its development, the Single Category Implicit Association Test (SC-IAT) [40]. The latter was administered in the current study to measure the participants’ implicit attitude towards food technology. Participants were asked to categorize stimuli belonging to the target category (food technology) and stimuli belonging to two opposite attribute categories (Positive and Negative), using the keyboard keys “A” and “L.” In two subsequent steps, the target category and one attribute category (e.g., Positive) share the same response key (step 1); then the target category and the other attribute category (e.g., Negative) share the same response key (step 2). A longer reaction time indicates that for the respondent it is more difficult to associate the target and attribute category (e.g., food technology and positive), whereas a shorter reaction time means that the two categories are easily associated. 

The stimuli in the attribute category were obtained by a pilot study in which 50 undergraduate students were asked to associate five adjectives to “food technology.” The ten most frequent adjectives were associated with their antonyms and used in the SC-IAT as the stimuli of the attribute category (e.g., “moral” and “immoral,” “useful” and “useless”). For the target category, the stimuli used were the following words: GMO, Nanotechnology, Coloring, Additive, Preservative, Sterilizing, Thickening. The presentation of the combination of target and attribute categories was counterbalanced, so that half of the participants were presented with Food technology and Positive first, and the other half with Food technology and Negative first. The final index was obtained by subtracting the participants’ time latency in the Food technology and Negative step from the participants’ time latency in the Food technology and Positive step (correcting for individual standard deviation; see [41]), so that positive values indicate a positive implicit attitude about food technology, whereas negative values indicate a negative implicit attitude. Tested for reliability, the SC-IAT proved adequate (α = 0.71).

The Food Technology Neophobia Scale (FTNS) was used to measure attitudes toward food technology [42,43,44]. In our study, the following subscales were used: 1. New food technologies are unnecessary (FTNS1); 2. Perception of risk (FTNS2); 3. (FTNS3) Healthy choices. Participants indicated their agreement with the statements on a 7-point scale from 1 (totally disagree) to 7 (totally agree). The three subscales proved to have adequate internal consistency (α_FTNS1_ = 0.80; α_FTNS2_ = 0.71; ρ_FTNS3_ = 0.73). Items were averaged in single scores. For FTNS1, the higher the score, the more negative the attitude; in the case of FTNS2 the higher the score, the more negative the perception of risk, and finally, for FTNS3 the higher the score, the more positive the perception of benefit. 

Participants also completed the Trust in Science Scale (TISS), a six items scale that focuses on public attitudes toward controversial scientific research and technologies [45]. Participants indicated their agreement on a scale from 1 to 5 (α = 0.69). Items were averaged in a single score (the higher the score, the higher the trust in science).

To measure social desirability (SD), the short form of the Marlowe-Crowne Social Desirability scale [46] was used. Answers were collected on a 1 to 6 scale (α = 0.67). Items were averaged in a single score (the higher the score, the higher the social desirability).

Participants’ self-reported consumption frequency of tomato products (FREQ) was measured by a single item, with the following response format: 1 = rarely or never; 2 = 1 or 2 times a week; 3 = several times a week; 4 = daily. 

Participants were also asked to self-report their level of knowledge about lycopene (KNOW) through a single item, with a 1 to 6 response scale. The higher the score, the higher the perceived level of knowledge about lycopene. Finally, SHOCK is a dummy variable which assumes value 0 in the treatment without information and value 1 in the following treatment with information about lycopene. Table 2 provides the summary statistics.

### 2.3. Experimental Design and Procedure

Several sessions of experimental auctions were conducted in the computer lab of the Department of Agricultural Sciences in Portici (Naples) in order to assess the willingness to pay (WTP) for a specific functional product (crushed tomatoes enriched with lycopene). The experimental design was computer administered in order to accelerate data acquisition and to minimize the possibility of error in the collecting of the data. The software were:

- *Z-tree* [47], for the collection of bids in auctions;
- *Google Drive*, to administer the questionnaires;
- *Millisecond Inquisit*, for the administration of SC-IAT.

For this experiment, the fifth-price mechanism with a full bidding process was employed. Currently, a fifth-price auction is considered an incentive-compatible mechanism [29]. In addition, the fifth-price mechanism represents an attempt to combine the advantages of the second-price mechanism and the random n^th^-price mechanism. Therefore, using the fifth-price mechanism could engage bidders with values on both tails of the value distribution [23]. We used the full bidding process instead of endowment bidding to eliminate any aversion to loss and risk exchanging of the participants [29,48]. Following Drichoutis, Lazaridis and Nayga [49], Bernard and He [50], and Hellyer, Fraser and Haddock-Fraser [48], we did not use the reference price, since we were aware of the possibility of bid affiliation. No price feedback among multiple rounds was reported [51]. 

The experiment is divided into several stages. During the experiment each participant is asked to use an ID (identifier) in order to trace the source computer of the data, thereby preserving complete anonymity.

At first, the SC-IAT is administered. After that, the experimenter provides participants with all of the information on the auction mechanism. Any communication between the subjects during the auction is prevented. The subjects are informed about the dominant strategy to reveal their true value for the products offered. To understand the bidding behavior and the mechanism, two training rounds are conducted using three different candy bars.

The products in the auction are two packs of three 400-gram cans of crushed tomatoes: Conventional crushed tomatoes, and crushed tomatoes enriched in lycopene (50% more). During the auction, each participant is asked to submit simultaneously a bid for each of the two crushed tomato products. The bids are collected, and the step is repeated in four additional rounds. In order to avoid any ordering effect, the products are simultaneously shown to the participants, and the order of the bids for each product is randomly assigned.

At the end of the five rounds, participants are informed that a second part of the experiment would start in a few minutes. Each participant is given a brief note on the nature of lycopene and the potential positive effects on human health (see Appendix). The information note is written considering the results produced by scientific research. 

After reading the text, that is the informative shock, the participants take part in a further non-hypothetical auction that takes place in the same conditions as the previous one. 

Immediately after the post-information auctions, participants complete the Food Technology Neophobia Scale (FTNS), the Trust in Science Scale (TISS), the Marlowe-Crowne Social Desirability scale, and the items about the frequency with which they consume tomato products and about their knowledge of lycopene.

Finally, a first random draw determines which auction is chosen (with or without informative shock), a second draw determines the selected round; finally, a random draw defines which of the crushed tomato products is selected. The top four bidders on the bidding product of the selected auction and round purchase the crushed tomato package, paying a price equivalent to the fifth-highest bid for the product. 

Basic socio-demographic characteristics are collected before thanking and de-briefing participants.

## 3. Statistical Analysis

### 3.1. Quantile Regression

The OLS analysis is complemented by the estimates of the regression at various quantiles. While OLS computes the regression at the mean, the quantile regression estimator [52,53] allows the impact of the explanatory variables to be investigated at different levels of the dependent variable: Not only at the center, the mean or the median, but also in the tails of the distribution, such as the first and third quartiles of the conditional distribution of bids. This is particularly relevant in the case of asymmetry, as is the case in the bid distributions.

For instance, in the linear regression model *y_i_* = $x_{i}{}^{’}$*β* + *e_i_*, where *x_i_* is the row vector including the i^th^ observation for all the explanatory variables of the model, the quantile regression objective function at the selected quantile *θ* is:

$$\underset{i}{\sum }\{\theta -1(y_{i}\leq x_{i}\beta )\}\left|y_{i}-x_{i}{}^{’}\beta \right|$$

where the absolute value of the regression errors, $\left|e_{i}\right|=\left|y_{i}-x_{i}{}^{’}\beta \right|,$ is asymmetrically weighted by *θ* or (1 − *θ*). The weights set the position of the estimated line and allow the estimated line to move away from the mean of the conditional distribution. For instance, the 10^th^ quantile regression partitions the residuals 10% below and 90% above the estimated regression, based on weights of *θ* = 0.10 assigned to the positive residuals and (1 − *θ*) = 0.90 to the negative ones. This determines the prevalence of negative errors, thereby locating the regression line in the lower tail, precisely at the 10^th^ quantile. At the median the weights are symmetric, *θ = 1 –* *θ = 0.5* and the objective function simplifies into $0.5\sum \left|y_{i}-x_{i}{}^{’}\beta \right|.$ Just as the sample median equally divides the observations, the median regression equally splits the regression residuals half above and half below the estimated equation [53].

### 3.2. Decomposition Analysis

To analyze a data set split in two different subsets, each identified by an index assuming values 0, the bid experiments before information on lycopene, and 1 otherwise, a decomposition approach can be implemented. On average, the Oaxaca [54] and Blinder [55] decomposition writes the difference between these subsets as:

E (y_1_ − y_0_) = E[*β*_1_ x_1_ − *β*_0_ x_0_ ] = E[*β*_1_ x_1_ − *β*_0_ x_1_ + *β*_0_ x_1_ − *β*_0_ x_0_] = E[(*β*_1_ − *β*_0_) x_1_ + *β*_0_ (x_1_ − x_0_)] = E(y_1/1_ − y_0/1_ + y_0/1_ − y_0/0_) (1)

where E(y_1/1_) = *β*_1_E(x_1_) and E(y_0/0_) = *β*_0_E(x_0_) are the realizations of the dependent variable within each subset, while the term E(y_0/1_) = *β*_0_E(x_1_), defined by the covariates of subset 1 evaluated at the coefficients of subset 0, represents the counterfactual, and cannot be observed. Counterfactual distributions are the result of a change in covariates, or a change in their relationship with the dependent variable, the regression coefficients. In the bids example y_1/1_ coincides with bids after the shock and y_0/0_ coincides with bids before the shock. They are respectively the observed bids in each subset, while y_0/1_ provides the subset 1 covariate, after shock, multiplied by subset 0 coefficients, pre-shock. The first term of the decomposition, y_1/1_ − y_0/1_, measures the difference in bids due to changes in the regression coefficients, (*β*_1_ − *β*_0_). The second term, instead, looks at the difference in bids due to changes in the covariates between subsets, and provides a measure of the composition effect. In this analysis the focus is on the coefficients effect since the bids are provided by the same individuals, so there is no difference in the covariates. The terms in the decomposition are computed at their average values,

Ey_1_ − Ey_0_ = (*β*_1_ − *β*_0_) Ex_1_ + *β*_0_ (Ex_1_ − Ex_0_ ) (2)

where expectations are replaced by their sample average and the parameters *β*_1_, *β*_0_ by their OLS estimates. The result is an average measure of bids difference between the two subsets. We focus on the behavior of (*β*_1_ − *β*_0_) Ex_1_, that would measure for each coefficient the impact of the informative shock on the bids for enriched and for conventional tomatoes.

### 3.3. Censoring

Since WTP cannot be negative, we also consider the Powell [56] censored least absolute deviations estimator (CLAD) and the bootstrap estimates of its sampling variance. The CLAD estimator is a generalization of the least absolute deviations (LAD) estimator, that is, the median regression. Unlike the standard estimators of the censored regression model, such as Tobit or other maximum likelihood approaches, the CLAD estimator is robust to heteroscedasticity and is consistent and asymptotically normal for a wide class of error distributions. Bootstrap estimates of the sampling variance are provided using a simple random design. The objective function of the Powell estimator is given by Σ|y_i_ − max (0; x_i_’*β*)| and is iteratively estimated.

## 4. Results

### 4.1. The Model

The model compares the WTP for the enriched and the conventional tomatoes as a function of three main groups of variables: Socio-demographic, attitudinal and control variables. The summary statistics of all the study variables are collected in Table 1, Table 2 and Table 3.

**Figure 1:**
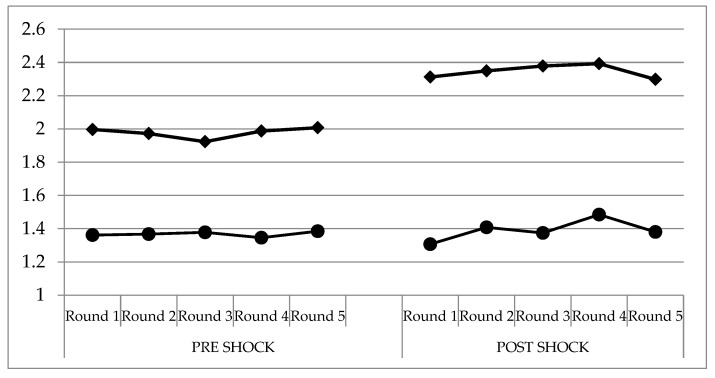
The Effect of Information on Mean Bids by Product and by Round. Note: the y axis is expressed in Euros (€).

In order to evaluate if information on the benefit of lycopene affects the consumers’ WTP both on canned tomatoes enriched with lycopene and conventional canned tomatoes (not enriched), we started with the comparison of the patterns of the bids before and after the informative shock (SHOCK in the tables) across the five rounds and for each product, as reported in Table 4 and Figure 1. 

**Figure 2:**
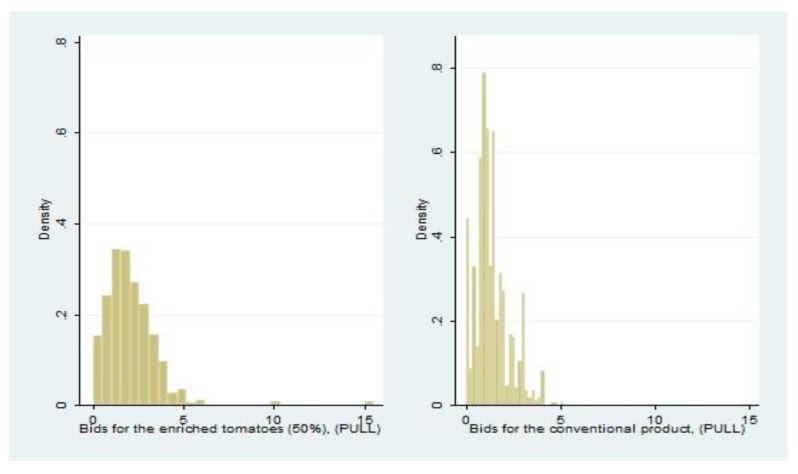
Empirical distributions of bids for the enriched and the conventional product.

Informative shock increases the bids for the enriched product, whereas it does not affect their level (mean) for the conventional one, although it affects the dispersion of the average bids. Figure 2 displays the histograms of the dependent variables, the bids for the enriched and the conventional product. The left graph is more dispersed than the one depicting the conventional product, and its sample mean is higher. Both distributions are characterized by skewness, that can be related to the presence of a few large values in the upper tail of the distributions, particularly in the bids for the enriched product. 

The second specific objective of the present research was to evaluate whether and how several selected variables, namely socio-demographics, attitudinal constructs and control variables, affect WTP. To pursue the objective two quantile regressions were estimated both for the conventional and the enriched products. The estimates of the regression coefficients are reported in Table 5 for the enriched product and in Table 6 for the conventional tomatoes. The last two columns of Table 5 and Table 6 report the OLS estimated coefficients explaining average bids for the enriched and the conventional tomatoes as a function of the selected variables. 

The results for the first, second and third quartile regression are reported in the first three columns of Table 5 and Table 6. Across quartiles the estimated coefficients do change, and depending on the selected quantile, the explanatory variables have a different impact on the dependent variable, a different explanatory power.

The results of the two regressions offer further evidence about the positive effect of the information about the benefit of lycopene on consumers’ WTP. Indeed, the informative shock (SHOCK) is statistically relevant at all quantiles and at the conditional mean/OLS in the regression for the enriched product of Table 5. Vice versa, for the conventional product this same variable is not statistically different from zero, as reported in Table 6. With regard to the role played by the selected socio-demographic variables, gender exerts a positive impact on bids both for the enriched and conventional product, while age and income are mostly not significant.

Overall, attitude toward food technologies exerts a key role in determining bids. The results of the two regressions for the conventional and the enriched canned tomatoes (Table 5 and Table 6) outline the different role of technophobia in the case of more or less familiar technology. In fact, in the case of conventional crushed tomatoes, which represent a very familiar food product generated by a mature technology, the perception of risks (FTNS2) is significant only at the 75^th^ percentile. In the case of enriched crushed tomatoes, that is an innovative and less familiar food product, the perception of risks is always statistically significant at all quantiles (see also [44]). Moreover, although canned tomatoes are considered by consumers as the result of mature and well-known technologies, it is confirmed that the implicit attitude (SC-IAT) reduces WTP for canned tomato pulp. Finally, the two regressions seem to reserve a minor role for self-reported previous knowledge. In fact, previous knowledge does not seem to play any role in the case of enriched products [57].

Both explicit (FTNS) and implicit attitudes (SC-IAT) are significant predictors, particularly at the upper quartile. As expected, the perception of risks linked to technologies decreases WTP while the perception of benefits increases it. The effect of implicit attitude is statistically significant and increases across quantiles. Although significant in the conventional product, the self-reported knowledge provides small estimated coefficients.

From a marketing point of view, this set of results highlights the existence of different consumers’ clusters. The clustering suggests the possibility of a fruitful communication and price strategy to build a market segmentation.

### 4.2. Results of the Decomposition Analysis

The third and final specific objective of the paper was to explore if and how the selected variables affect the possible change in prices after the informative shock; that is, assessing the moderator role of the variables on the WTP. 

To this end a decomposition approach is implemented and next the relevant variables signaled by the decomposition are added to the main model as interaction terms. This allows us to measure the relevance of their additional impact.

The variable SHOCK is a dummy, assuming a zero value before, and unit value after the informative talk on the qualities of lycopene. The dummy measures the additional explanatory power of information on bids, net of the effect of the informative talk on all the other variables of the equation. It is however possible that the informative talk has affected bids not only directly, but also indirectly, through its impact on some/all the other explanatory variables. In order to check if this is the case, we implemented a decomposition analysis.

Table 7 reports the results of the decomposition. In this table the left columns report the decomposition of the enriched product and the right columns those of the conventional product. The top section of the table reports the overall average decomposition, while the bottom section of the table considers the impact of the informative shock on each regression coefficient as computed on average, by OLS. In the top section, the comparison between bids before and after the informative shock in the enriched product, as computed on average, yields a total difference Ey_1_ − Ey_0_ = 0.369 that is statistically different from zero. The latter is split into the endowment effect (Ex_1_ − Ex_0_), that is equal to zero, since people characteristics do not change after the informative shock, and the coefficient effect that is equal to the total difference, (*β*_1_ − *β*_0_) Ex_1_ = 0.369. Therefore, there is a significant difference between bids before and after the shock in the enriched product case that cannot be explained by the covariates of the model but must be credited to information. For the conventional product instead, the overall average difference in bids before and after the shock is not statistically relevant.

Next the impact of the shock on each explanatory variable is reported in the lower section of this table. For the enriched product the informative shock has a relevant impact on the age coefficient, and on the knowledge of the qualities of lycopene. After the briefing on lycopene qualities, the age coefficient increases, while the knowledge coefficient decreases the impact on bids. Vice versa, in the conventional product the knowledge of lycopene properties and age have no relevant impact on any of the variables explaining bids.

### 4.3. Censoring

Unlike the standard estimators of the censored regression model such as Tobit or other maximum likelihood approaches, the CLAD estimator is robust to heteroscedasticity and is consistent and asymptotically normal for a wide class of error distributions. Bootstrap estimates of the sampling variance are provided using a simple random design. The objective function of the Powell estimator, given by Σ|y_i_ − max (0; x_i_’β)|, is iteratively estimated. Table 8 reports the results. For comparison’s sake, the table reports the results of the uncensored median regression (LAD in the table) and of the mean regression (OLS in the table) estimators.

We observe that, adding the interactions Shock*Age and Shock*Know, the main effect of the shock variable drops in significance. The negative sign of the know*shock coefficient means that individuals with a low level of previous knowledge are characterized by higher bids as a result of the informative shock. The relevant role played by attitude variables towards technologies, which already emerged in the quantile regression analysis on functionalized products, is fully confirmed.

The LAD estimated coefficients are numerically identical to CLAD results, the sole difference is in the estimated standard errors. The OLS results are not numerically identical to the CLAD estimates, however they fully confirm the CLAD findings.

## 5. Discussion

The present paper examined the following research questions. First, we evaluated whether information on the benefit of lycopene affects the consumers’ WTP both on canned tomatoes enriched with lycopene and conventional canned tomatoes (not enriched). Secondly, we studied whether and how several selected variables, namely socio-demographics, attitudinal constructs and control variables, affect WTP. Finally, we explored whether the selected variables affect the possible change in prices after the informative shock, which means assessing the moderator role of the variables on the WTP. 

### 5.1. Premium Price for Lycopene-enriched Canned Tomato

From our results emerges the higher willingness to pay for the enriched product compared to the conventional one. This premium price that participants are willing to pay for the functionalized product has been confirmed in all five rounds. 

This means that, even without any specific information about the beneficial properties of lycopene, the WTP changes, due to the label on the package of the enriched product highlighting the higher content of lycopene.

The higher WTP for the functionalized products confirms previous results obtained in the case of other food products such as bread and yogurt [48,57]. However, it is important to stress that, differently from previous research, in our study the functional attribute is naturally held both in the conventional and in the enriched product. The higher quantity of lycopene of the functionalized product is determined by the use of specific tomato varieties identified by genomic research and characterized by a higher content of lycopene. Second, previous research used claims focusing on the beneficial health effect of the functionalized product. Therefore, participants’ evaluation of the functionalization itself, and of the healthiness of the attribute in question, are not distinguishable. On the contrary, our study was designed to allow the investigation of participants’ attitudes and behavioral reaction to functionalization itself, and the relevance of the attribute is particularly related to the functionalization, which was communicated through an information shock. However, the effect of the information is limited only to the case of lycopene-enriched products, whereas no difference in bids emerges for the conventional product either on average or at the quantiles. A possible explanation of these results could be that the label of the functionalized product, which communicates the higher content in lycopene, increased the salience of this attribute.

### 5.2. Effect of Attitudinal and Socio-demographic Variables

The second issue discusses the premium price that participants are willing to pay for the enriched product as influenced by the explanatory variables. First of all, a significant role of socio-demographic variables emerges. As shown in Table 5 and Table 6, women are characterized by a higher WTP both in the conventional and lycopene-enriched case. Income is not significant at any of the quantiles considered. The influence of age is never significant for the conventional crushed tomatoes, whereas in the case of the enriched products it shows a positive and significant coefficient both on average and at the median. Finally, a higher frequency of consumption is associated with a higher WTP. 

The quantile regression on functionalized products shows the importance of attitudinal variables. The set of variables used to measure the degree of aversion/favor towards food technologies contribute to determine the WTP for the functionalized product. While FTNS1, which measures the perceived un-usefulness of new food technology, is never statistically significant, the perception of the risks associated with the use of food-related technologies (FTNS2) has a negative effect on WTP, which is more pronounced in the case of the enriched products. The role played by the perception of benefits (FTNS3) becomes more relevant in the case of higher WTP, since the coefficient increases moving from the median to the 75^th^ percentile. The role of the FTNS three dimensions is in line with previous results in the literature considering different food products and food categories [42]. The importance of the attitude towards technologies is also confirmed by the significant role played by implicit attitude, measured through SC-IAT [13,38]. Finally, to complete the set of indicators used to capture the effects of technophobia, the degree of trust towards science also affects the WTP for enriched tomatoes: Individuals with higher confidence in scientific research show a higher WTP. TISS does not exert any effect in the case of more familiar products like the conventional canned tomatoes. To the best of our knowledge, this is the first study that shows the relevance of explicit and implicit attitudes towards food technology on people’s WTP in a non-hypothetical experiment. Findings also suggest that, at least in the case of the cluster of consumers with the higher WTP, it may be reasonable to build communicative messages and market segmentation drawing on health benefits associated with functionalized food products [58].

### 5.3. The Role of Information and Knowledge on WTP for Lycopene-Enriched Canned Tomato

Referring to the last issue, assessing the moderator role of the variables on the WTP, the Oaxaca decomposition suggests a potential role of moderator for previous knowledge and age: When the level of knowledge is low, the informative shock produces greater effects in terms of premium price, while the informative shock is associated with higher bids for the enriched product as people grow older. The CLAD regression allows the effects of the informative shock on bidding to be analyzed in a censoring context to account for 0 bids (Table 8). The CLAD regression is also carried out considering the interactions terms Shock*Knowledge and Shock*Age suggested by the results of the Oaxaca decomposition. The shock variable is no longer significant when adding interactions; therefore, the variables selected to explain the divergence in the bid before and after the informative shock completely explain the phenomenon. The role of information is already well-known, and recently confirmed also in the case of very familiar functionalized food products characterized by an image of naturalness such as apples [59]. However, to the best of our knowledge, this research is the first attempt to identify the factors that are involved in the formation of the premium price resulting from an information shock. The CLAD regression carried out without interaction confirms the results already discussed for the regression with interactions, because in this case the dummy variable on the treatment is significant, confirming the importance of the level of previous knowledge as a moderator of the effect of the informative shock on bids. The OLS regressions confirm these results. Finally, since we can interpret the administration of the information shock as an increase in nutritional knowledge, our results also confirm the evidence recently produced by Steinhauser and Hamm [60] on the positive effect of nutritional knowledge on the intention to purchase.

### 5.4. Limitation of the Study and Further Research

Turning to the implementation of the experimental auction, there are some limitations that have to be acknowledged. First the nature of the sample, composed by university students who may have a starting degree of knowledge about lycopene different from that of the general population, and they maybe also have a different attitude towards food technologies. It is up to future research to widen the study on the topic of food functionalization involving more heterogeneous samples. Second, the within treatment, which allows us to trace the effect of the information in reference to each participant, could also generate in the participants anchoring effects, and could amplify problems of social desirability, thus introducing some bias in the estimates. Therefore, another intriguing path for further research may be the study of WTP for functionalized food products with between-subject treatments. Finally, the explicit and implicit attitudinal variables we use in the study, which showed a significant role, are cognitive in nature. An important development of the study about functionalization should include the emotional dimension of evaluation [61,62].

## 6. Conclusions

In the above analysis we investigated three main issues: Is information on the benefit of lycopene relevant to determine consumers’ WTP? What are the main factors affecting the WTP? Are there variables assuming a moderator role on the WTP?

—The informative shock is found relevant in defining consumer willingness to pay both on average and in the tails of WTP distribution. 

—WTP for functionalized canned tomatoes is strongly affected by attitudes toward technology. Indeed, both explicit and implicit attitudes have a relevant and significant impact on WTP, together with the trust in science. 

—Finally, the moderator role of knowledge on WTP has been ascertained. People with little knowledge on lycopene properties increase their willingness to pay after the informative shock. 

The quantile regression approach implemented throughout the paper has pointed out a changing impact of each variable along the WTP distribution. From a marketing point of view this analysis shows the existence of different consumers’ clusters. This suggests implementing different communication and price strategies to build a market segmentation.

## Figures and Tables

**Table 1:** Summary statistics of the explanatory variables of the model (*n* = 100).

Socio-Demographic	Mean	Std. Dev.	q25	q50	q75
Age	23.88	4.00	21.0	24.0	25.5
Gender	0.560	0.49	0.00	1.00	1.00
Income	2.300	0.87	2.00	2.00	3.00

**Table 2:** Summary statistics of the Control variables and measures.

**Control Variables**	**Mean**	**Std. Dev.**	**q25**	**q50**	**q75**
Consumption Frequency (FREQ)	2.880	0.58	3.00	3.00	3.00
Lycopene Knowledge (KNOW)	2.810	2.87	0.00	2.50	6.00
**Measures**	**Mean**	**Std. Dev.**	**q25**	**q50**	**q75**
SC_IAT	−0.068	0.41	−0.40	−0.09	0.25
Social Desirability (SD)	3.975	0.65	3.61	4.00	4.33
FTNS Unnecessary (FTNS1)	3.445	1.12	2.50	3.33	4.33
FTNS Risks (FTNS2)	4.040	1.19	3.37	4.00	4.75
FTNS Benefits (FTNS3)	3.185	1.28	2.50	3.00	4.00
Trust in Science (TISS)	2.31	0.49	1.80	2.20	2.40
**Treatment Variable**	**Mean**	**Std. Dev.**	**q25**	**q50**	**q75**
SHOCK	0.500	0.50	0.00	0.50	1.00

**Table 3:** Summary statistics of the dependent variables.

Dependent Variable	Mean	Std. Dev.	q25	q50	q75
BIDL50 (*n* = 1000)	2.161	1.58	1.20	1.90	2.80
BIDCONV (*n* = 1000)	1.378	0.87	0.80	1.20	1.80
BIDL50, SHOCK = 0 (*n* = 500)	1.977	1.39	1.05	1.80	2.70
BIDCONV, SHOCK = 0 (*n* = 500)	1.367	0.88	0.80	1.20	2.70
BIDL50, SHOCK = 1 (*n* = 500)	2.346	1.74	1.31	2.10	3.00
BIDCONV, SHOCK = 1 (*n* = 500)	1.390	0.87	0.80	1.20	1.80

Note. BIDL50 = bids for the enriched product; BIDCONV = bids for the conventional product; SHOCK = treatment variable that assumes value 0 before the information shock, and value 1 after the information shock; *n* = number of observations.

**Table 4:** Summary of Mean and Median (in parenthesis) willingness to pay (WTP) by Products and Treatment.

	Pre-Shock		Post Shock	
	Conventional	Enriched	Conventional	Enriched
1st Round, *n* = 100	1.36 (1.20)	2.00 (1.75)	1.31 (1.20)	2.31 (2.05)
2nd Round, *n* = 100	1.37 (1.22)	1.97 (1.80)	1.41 (1.20)	2.35 (2.10)
3rd Round, *n* = 100	1.38 (1.20)	1.92 (1.79)	1.37 (1.20)	2.38 (2.05)
4th Round, *n* = 100	1.35 (1.20)	1.99 (1.90)	1.48 (1.30)	2.39 (2.10)
5th Round, *n* = 100	1.38 (1.20)	2.01(1.85)	1.38 (1.20)	2.30 (2.00)

**Table 5:** OLS and quantile regressions for the enriched tomatoes (*n* = 1000).

	q25		q50		q75		OLS	
Variables	B	SE	B	SE	B	SE	B	SE
AGE	−0.018	0.013	0.036 **	0.012	0.021	0.021	0.099 **	0.011
GENDER	0.457 **	0.116	0.398 **	0.105	0.432 *	0.188	0.722 **	0.102
INCOME	−0.014	0.066	−0.020	0.060	0.025	0.108	−0.004	0.059
FREQ	0.143	0.092	0.285 **	0.084	0.586 **	0.150	0.498 **	0.082
FTNS1	−0.041	0.057	0.024	0.051	−0.149	0.092	−0.064	0.050
FTNS2	−0.145 **	0.056	−0.247 **	0.051	−0.234 *	0.092	−0.187 **	0.050
FTNS3	−0.003	0.044	0.146 **	0.040	0.326 **	0.072	0.241 **	0.039
SD	0.041	0.085	0.065	0.077	0.243	0.138	0.182 *	0.075
TISS	0.172	0.114	0.234 *	0.104	0.627 **	0.186	0.362 **	0.101
SC-IAT	0.223	0.131	0.583 **	0.119	0.381	0.214	0.418 **	0.116
SHOCK	0.301 **	0.101	0.2 *	0.092	0.403 *	0.164	0.369 **	0.089
KNOW	−0.004	0.018	0.017	0.016	−0.002	0.030	−0.038 *	0.016
constant	1.02	0.618	−0.298	0.561	−1.78	1.00	−3.37 **	0.549

Note: Dependent variable: Bids for the enriched product. B = estimated coefficients; SE = Standard Error. * = *p* < 0.05; ** = *p* < 0.01.

**Table 6:** Estimated regressions for the conventional product (*n* = 1000).

	q25		q50		q75		OLS	
Variables	B	SE	B	SE	B	SE	B	SE
AGE	0.004	0.008	0.006	0.007	−0.011	0.014	−0.004	0.007
GENDER	0.421 **	0.074	0.245 **	0.065	0.498 **	0.124	0.377 **	0.060
INCOME	−0.070	0.043	−0.120 **	0.037	0.101	0.071	−0.000	0.035
FREQ	0.040	0.059	0.007	0.052	0.185	0.099	0.124 **	0.048
FTNS1	0.015	0.036	0.002	0.032	0.052	0.061	−0.018	0.029
FTNS2	−0.028	0.036	−0.048	0.032	−0.157 *	0.060	−0.083 **	0.029
FTNS3	0.067 **	0.028	0.134 **	0.025	0.216 **	0.047	0.093 **	0.023
SD	−0.002	0.054	0.085	0.048	0.081	0.091	0.030	0.044
TISS	−0.182 *	0.073	−0.182 **	0.065	0.182	0.123	0.072	0.060
SC-IAT	0.147	0.084	0.119	0.075	0.269	0.141	0.214 **	0.069
SHOCK	−0.016	0.065	−0.004	0.057	0.030	0.108	0.023	0.053
KNOW	0.024 *	0.011	0.055 **	0.010	0.038 *	0.019	0.025 **	0.009
constant	0.702	0.398	0.908 *	0.351	−0.046	0.662	0.661 *	0.324

Note: Dependent variable: Bids for the enriched product. B = estimated coefficients; SE = Standard Error. * = *p* < 0.05; ** = *p* < 0.01.

**Table 7:** Oaxaca-Blinder decomposition of the total difference due to information (*n* = 1000).

**Functional**			**Conventional**		
mean group_0	1.98	0.062	mean group_0	1.37	0.040
mean group_1	2.35	0.078	mean group_1	1.39	0.039
mean difference	0.369 **	0.100	mean difference	0.023	0.056
in endowments	0	0.054	in endowments	0	0.044
in coefficients	0.369 **	0.091	in coefficients	0.023	0.035
**Variable**	**B**	**SE**	**Variable**	**B**	**SE**
AGE	1.74 **	0.56	AGE	0.007	0.34
GENDER	0.094	0.11	GENDER	−0.065	0.06
INCOME	−0.018	0.26	INCOME	−0.040	0.16
FREQ	0.114	0.46	FREQ	−0.226	0.28
KNOW	−0.241 **	0.09	KNOW	−0.004	0.05
FTNS1	0.350	0.34	FTNS1	0.056	0.20
FTNS2	−0.226	0.40	FTNS2	−0.148	0.24
FTNS3	0.290	0.25	FTNS3	−0.068	0.15
SD	0.559	0.59	SD	0.315	0.35
TISS	−0.182	0.42	TISS	−0.105	0.25
SC_IAT	0.005	0.01	SC_IAT	0.001	0.01
Constant	−2.112	1.08	Constant	0.299	0.64

Note: Dependent variable: Bids for the enriched and conventional products. B = estimated coefficients; SE = Standard Error, ** = *p* < 0.01.

**Table 8:** Censored and uncensored regression with (Model 1) and without (Model 2) interactions (*n* = 1000).

				Model 1						Model 2		
Variable	LAD	SE	CLAD	SE	OLS	SE	LAD	SE	CLAD	SE	OLS	SE
SHOCK	0.262	0.555	0.262	0.518	−1.079	1.266	0.200 *	0.092	0.200 *	0.080	0.369 **	0.089
INCOME	−0.006	0.060	−0.006	0.125	−0.004	0.052	−0.020	0.060	−0.020	0.124	−0.004	0.051
AGE	0.035 *	0.017	0.035 *	0.015	0.063 *	0.031	0.036 **	0.012	0.036 **	0.013	0.099 **	0.029
GENDER	0.392 **	0.105	0.392 **	0.015	0.722 **	0.109	0.398 **	0.105	0.398 *	0.157	0.722 **	0.110
FREQ	0.299 **	0.084	0.300 **	0.071	0.498 **	0.085	0.285 **	0.084	0.285 **	0.081	0.498 **	0.086
FTNS1	0.005	0.051	0.005	0.073	−0.064	0.043	0.024	0.051	0.024	0.075	−0.064	0.043
FTNS2	−0.252 **	0.051	−0.252 **	0.049	−0.186 **	0.043	−0.247 **	0.051	−0.247 **	0.049	−0.186 **	0.043
FTNS3	0.175 **	0.040	0.175 **	0.052	0.241 **	0.053	0.146 **	0.040	0.146 **	0.044	0.241 **	0.054
TISS	0.329 **	0.104	0.330	0.226	0.362 **	0.096	0.234 *	0.104	0.234	0.210	0.362 **	0.095
SD	0.169*	0.077	0.170	0.148	0.181 *	0.073	0.065	0.077	0.065	0.152	0.181 *	0.074
SC-IAT	0.661 **	0.119	0.661 **	0.193	0.417 **	0.112	0.583 **	0.119	0.583 **	0.175	0.417 **	0.112
KNOW	0.062 **	0.023	0.062 *	0.029	0.009	0.022	0.017	0.017	0.017	0.024	−0.038 *	0.016
Shock*Know	−0.089 **	0.032	−0.089 **	0.023	−0.094 *	0.036						
Shock*Age	0.008	0.023	0.008	0.023	0.072	0.057						

*Note*: Dependent variable: Bids for the enriched product. B = estimated coefficients; SE = Standard Error. * = *p* < 0.05; ** = *p* < 0.01.

## Appendix A. Informative Shock

Lycopene is a natural element which can be found in some plant origin food. The major source of lycopene is represented by the tomato. It has been shown that lycopene has a high antioxidant capacity that neutralizes free radicals, protecting cells from the damage that these reactive elements can cause. Lycopene also reduces the oxidation of lipids, and in particular acts against the oxidation of cholesterol. Another possible application of lycopene is represented by the protective action it exerts on the skin in case of long exposure to UV rays. Finally, it has been highlighted that lycopene can inhibit the growth of cancer cells and the most recent studies show that the weekly consumption of seven or more portions of tomatoes leads to a reduction in cancer risk.

## References

[1] Regmi A., Gehlhar M.J. (2005). New Directions in Global Food Markets.

[2] Sadler J. (2005). Innovation in Functional Food and Drinks.

[3] Szakály Z., Szente V., Kövér G., Polereczki Z., Szigeti O. (2012). The influence of lifestyle on health behavior and preference for functional foods. Appetite.

[4] Peake H., Stockely M., Frost G. (2001). What nutritional support literature do hospital nursing staff require?. J. Hum. Nutr. Diet..

[5] Alzamora S.M., Salvatori D., Tapia M.S., López-Malo A., Welti-Chanes J., Fito P. (2005). Novel functional foods from vegetable matrices impregnated with biologically active compounds. J. Food Eng..

[6] Frewer L., Scholderer J., Lambert N. (2003). Consumer acceptance of functional foods: Issues for the future. Br. Food J..

[7] Heasman M., Mellentin J. (2001). The Functional Foods Revolution: Healthy People. Healthy Profits.

[8] Grunert K.G. (2010). European consumers’ acceptance of functional foods. Ann. NY Acad. Sci..

[9] Jousse F. (2008). Modeling to improve the efficiency of product and process development. Compr. Rev. Food Sci. Food Saf..

[10] Siro I., Kápolna E., Kápolna B., Lugasi A. (2008). Functional food. Product development, marketing and consumer acceptance—A review. Appetite.

[11] OCDA (2001). Estudio de Mercado. Informe Histórico Observatorio del Consumo y la Distribución Alimentaria.

[12] Bäckström A., Pirttilä-Backman A.M., Tuorila H. (2003). Dimensions of novelty: A social representation approach to new foods. Appetite.

[13] Furno M., Verneau F., Sannino G. (2016). Assessing hypothetical bias: An analysis beyond the mean of functional food. Food Qual. Prefer..

[14] IFIC (1999). Functional Foods: Attitudinal Research (1996–1999).

[15] de-Magistris T., López-Galán B., Caputo V. (2016). The impact of body image on the WTP values for reduced-fat and low-salt content potato chips among obese and non-obese consumers. Nutrients.

[16] Verbeke W. (2005). Consumer acceptance of functional foods: Socio-demographic, cognitive and attitudinal determinants. Food Qual. Prefer..

[17] Verbeke W. (2006). Functional foods: Consumer willingness to compromise on taste for health?. Food Qual. Prefer..

[18] La Barbera F., Amato M., Sannino G. (2016). Understanding consumers’ intention and behaviour towards functionalised food: The role of knowledge and food technology neophobia. Br. Food J..

[19] Temesi Á., Bacsó Á., Grunert K.G., Lakner Z. (2019). Perceived Correspondence of Health Effects as a New Determinant Influencing Purchase Intention for Functional Food. Nutrients.

[20] de-Magistris T., Del Giudice T., Verneau F. (2015). The effect of information on willingness to pay for canned tuna fish with different corporate social responsibility (CSR) certification: A pilot study. J. Consum. Aff..

[21] Dillaway R., Messer K.D., Bernard J.C., Kaiser H.M. (2011). Do consumer responses to media food safety information last?. Appl. Econ. Perspect. Policy.

[22] De Steur H., Buysse J., Feng S., Gellynck X. (2013). Role of Information on Consumers’ Willingness-to-pay for Genetically-modified Rice with Health Benefits: An Application to China. Asian Econ. J..

[23] Lusk J.L., House L.O., Valli C., Jaeger S.R., Moore M., Morrow J.L., Traill W.B. (2004). Effect of information about benefits of biotechnology on consumer acceptance of genetically modified food: Evidence from experimental auctions in the United States, England, and France. Eur. Rev. Agric. Econ..

[24] ISMEA (2017). I Numeri della Filiera del Pomodoro da Industria.

[25] Poojary M.M., Passamonti P. (2015). Extraction of lycopene from tomato processing waste: Kinetics and modelling. Food Chem..

[26] Takeshima M., Ono M., Higuchi T., Chen C., Hara T., Nakano S. (2014). Anti-proliferative and apoptosis-inducing activity of lycopene against three subtypes of human breast cancer cell lines. Cancer Sci..

[27] Kaur G., Verma N. (2015). Nature curing cancer–review on structural modification studies with natural active compounds having anti-tumor efficiency. Biotechnol. Rep..

[28] EFSA Panel on Dietetic Products, Nutrition and Allergies (NDA) (2011). Scientific Opinion on the substantiation of health claims related to lycopene and protection of DNA, proteins and lipids from oxidative damage (ID1608, 1609, 1611, 1662, 1663, 1664, 1899, 1942, 2081, 2082, 2142, 2374), protection of the skin from UV-induced (including photo-oxidative) damage (ID1259, 1607, 1665, 2143, 2262, 2373), contribution to normal cardiac function (ID1610, 2372), and maintenance of normal vision (ID1827) pursuant to Article 13(1) of Regulation (EC) No 1924/2006. EFSA J..

[29] Lusk J.L., Shogren J. (2007). Experimental Auctions: Methods and Applications in Economic and Marketing Research.

[30] Ballco P., de-Magistris T., Caputo V. (2019). Consumer preferences for nutritional claims: An exploration of attention and choice based on an eye-tracking choice experiment. Food Res. Int..

[31] Corrigan J.R., Rousu M.C. (2008). Testing whether field auction experiments are demand revealing in practice. J. Agric. Res. Econ..

[32] Chang J.B., Lusk L.J., Norwood B.F. (2009). How Closely Do Hypothetical Surveys and Laboratory Experiments Predict Field Behaviour?. Am. J. Agric. Econ..

[33] Depositario D.P., Nayga R., Wu X., Laude T.P. (2009). Should students be used as subjects in experimental auctions?. Econ. Lett..

[34] Greenwald A.G., Banaji M.R. (1995). Implicit social cognition: Attitudes, self-esteem, and stereotypes. Psychol. Rev..

[35] Greenwald A.G., Poehlman T.A., Uhlmann E.L., Banaji M.R. (2009). Understanding and using the Implicit Association Test: III. Meta-analysis of predictive validity. J. Personal. Soc. Psychol..

[36] La Barbera F., Verneau F., Amato M., Grunert K.G. (2018). Understanding Westerners’ disgust for the eating of insects: The role of food neophobia and implicit associations. Food Qual. Prefer..

[37] Verneau F., La Barbera F., Kolle S., Amato M., Del Giudice T., Grunert K.G. (2016). The effect of communication and implicit associations on consuming insects: An experiment in Denmark and Italy. Appetite.

[38] Verneau F., La Barbera F., Del Giudice T. (2017). The role of implicit associations in the hypothetical bias. J. Consum. Aff..

[39] Greenwald A.G., McGhee D.E., Schwartz J.L. (1998). Measuring individual differences in implicit cognition: The implicit association test. J. Personal. Soc. Psychol..

[40] Karpinski A., Steinman R.B. (2006). The single category implicit association test as a measure of implicit social cognition. J. Personal. Soc. Psychol..

[41] Greenwald A.G., Nosek B.A., Banaji M.R. (2003). Understanding and using the Implicit Association Test: I. An improved scoring algorithm: Correction to Greenwald et al. (2003). J. Personal. Soc. Psychol..

[42] Cox D.N., Evans G. (2008). Construction and validation of a psychometric scale to measure consumers’ fears of novel food technologies: The food technology neophobia scale. Food Qual. Prefer..

[43] Coppola A., Verneau F., Caracciolo F. (2014). Neophobia in food consumption: An empirical application of the FTNS scale in southern Italy. Ital. J. Food Sci..

[44] Verneau F., Caracciolo F., Coppola A., Lombardi P. (2014). Consumer fears and familiarity of processed food. The value of information provided by the FTNS. Appetite.

[45] Bak H.J. (2001). Education and public attitudes toward science: Implications for the “deficit model” of education and support for science and technology. Soc. Sci. Q..

[46] Reynolds W.M. (1982). Development of reliable and valid short forms of the Marlowe-Crowne Social Desirability Scale. J. Clin. Psychol..

[47] Fischbacher U. (2007). z-Tree: Zurich toolbox for ready-made economic experiments. Exp. Econ..

[48] Hellyer N.E., Fraser I., Haddock-Fraser J. (2012). Food Choice, Health Information and Functional Ingredients: An Experimental Auction Employing Bread. Food Policy.

[49] Drichoutis A.C., Lazaridis P., Nayga R.M. (2008). The role of reference prices in experimental auctions. Econ. Lett..

[50] Bernard J.C., He N. (2010). Confounded by the field: Bidding in food auctions when field prices are increasing. Agric. Resour. Econ. Rev..

[51] Corrigan J.R., Drichoutis A., Lusk J., Nayga R., Rousu M.C. (2012). Repeated Rounds with Price Feedback in Experimental Auction Valuation: An Adversarial Collaboration. Am. J. Agric. Econ..

[52] Koenker R., Bassett G. (1978). Regression quantiles. Econometrica.

[53] Koenker R. (2005). Quantile Regression.

[54] Oaxaca R. (1973). Male-female wage differentials in urban labor markets. Int. Econ. Rev..

[55] Blinder A.S. (1973). Wage discrimination: Reduced form and structural estimates. J. Hum. Resour..

[56] Powell J.L. (1984). Least absolute deviations estimation for the censored regression model. J. Econom..

[57] Vecchio R., Van Loo E.J., Annunziata A. (2016). Consumers’ willingness to pay for conventional, organic and functional yogurt: Evidence from experimental auctions. Int. J. Consum. Stud..

[58] Coppola A., La Barbera F., Verneau F. (2015). Fair trade products’ consumption: A market segmentation by personal values. Qual. Access Success.

[59] Wortmann L., Enneking U., Daum D. (2018). German consumers’ attitude towards selenium-biofortified apples and acceptance of related nutrition and health claims. Nutrients.

[60] Steinhauser J., Hamm U. (2018). Consumer and product-specific characteristics influencing the effect of nutrition, health and risk reduction claims on preferences and purchase behavior–A systematic review. Appetite.

[61] Amato M., Fasanelli R., Riverso R. (2019). Emotional profiling for segmenting consumers: The case of household food waste. Qual. Access Success.

[62] La Barbera F., Riverso R., Verneau F. (2016). Understanding beliefs underpinning food waste in the framework of the theory of planned behaviour. Qual. Access Success.

